# Draft genome sequence of *myo*-inositol utilizing *Aeromonas dhakensis* 1P11S3 isolated from striped catfish (*Pangasianodon hypopthalmus)* in a local fish farm in Malaysia

**DOI:** 10.1016/j.dib.2022.107974

**Published:** 2022-02-22

**Authors:** Mohamad Azzam-Sayuti, Md Yasin Ina-Salwany, Mohd Zamri-Saad, Salleh Annas, Mark R. Liles, Tingbi Xu, Mohammad Noor Azmai Amal, Mohd Termizi Yusof

**Affiliations:** a Aquatic Animal Health and Therapeutics Laboratory, Institute of Bioscience, Universiti Putra Malaysia, UPM 43400 Serdang, Selangor, Malaysia; b Department of Aquaculture, Faculty of Agriculture, Universiti Putra Malaysia, UPM 43400 Serdang, Selangor, Malaysia; c Department of Veterinary Laboratory Diagnosis, Faculty of Veterinary Medicine, Universiti Putra Malaysia, UPM 43400 Serdang, Selangor, Malaysia; d Department of Biological Sciences, Auburn University, Auburn, AL 36849, USA; e Department of Biology, Faculty of Science, Universiti Putra Malaysia, UPM 43400 Serdang, Selangor, Malaysia; f Department of Microbiology, Faculty of Biotechnology and Biomolecular Sciences, Universiti Putra Malaysia, UPM 43400 Serdang, Selangor, Malaysia

**Keywords:** Draft genome, *myo-*inositol, *A. dhakensis*, Malaysia

## Abstract

A hypervirulent pathotype of *A. hydrophila* (vAh) is responsible for Motile *Aeromonas* Septicemia (MAS) and causes mass mortalities among farmed carp and catfish species in the USA and China. One unique phenotype for vAh among other *A. hydrophila* strains is the ability to utilize *myo-*inositol as a sole carbon source. While screening for *Aeromonas* isolates from diseased fish that can grow using *myo-*inositol as a sole carbon source, *A. dhakensis* 1P11S3 was isolated from the spleen of striped catfish (*Pangasianodon hypopthalmus)* displaying clinical MAS symptoms from a freshwater farm in Malaysia. *Aeromonas dhakensis* is also an important pathogen in aquaculture, and in this study, we report the draft genome sequence for *A. dhakensis* 1P11S3, that utilize *myo-*inositol as a sole carbon source.

## Specifications Table

**Table:** 

Subject	Biology
Specific subject area	Microbiology, Genomics, Biotechnology
Type of data	Table, figures
How data were acquired	The draft genome sequence was processed using Illumina HiSeq instrument
Data format	Raw, analyzed and deposited
Parameters for data collection	*Aeromonas dhakensis* 1P11S3 was isolated from the spleen of cultured striped catfish (*Pangasianodon hypopthalmus)* in Malaysia. Genomic DNA extraction and sequencing were performed.
Description of data collection	*Aeromonas dhakensis* 1P11S3 was able to grow in M9 agar with *myo*-inositol as a sole carbon source. Genomic DNA was isolated from a pure culture of *A.dhakensis* 1P11S3
Data source location	*Aeromonas dhakensis* 1P11S3 was isolated from the spleen of cultured striped catfish (*Pangasianodon hypopthalmus)* in Tumpat, Kelantan, Malaysia. Latitude and longitude: 6.196106 N 102.148057 E
Data accessibility	Data are publicly available at NCBI GenBank https://www.ncbi.nlm.nih.gov/assembly/GCA_015666195.1 https://www.ncbi.nlm.nih.gov/biosample/SAMN16824286 https://www.ncbi.nlm.nih.gov/bioproject/PRJNA679132 https://www.ncbi.nlm.nih.gov/sra/?term=PRJNA679132
Related research article	M. Azzam-Sayuti, M.Y. Ina-Salwany, M. Zamri-Saad, M.T. Yusof, S. Annas, M.Y. Najihah, et al., The prevalence, putative virulence genes, and antibiotic resistance profiles of *Aeromonas* spp. isolated from cultivated freshwater fish in Peninsular Malaysia, Aquaculture 540 (2021) 736719. DOI: https://doi.org/10.1016/j.aquaculture.2021.736719

## Value of the Data

• The draft genome of *A. dhakensis* 1P11S3, isolated from cultured freshwater fish will be useful for further research on the influence of *myo-*inositol catabolism to the virulence of *A. dhakensis.*
• Data from *A. dhakensis* 1P11S3 will facilitate understanding of lateral gene transfer of *myo*-inositol catabolism genes
• Data on the genome sequence of *A. dhakensis* 1P11S3 can be used for comparative genomic studies with other *Aeromonas* spp. disease isolates, including vAh strains.
• Data on the genome sequence of *A. dhakensis* 1P11S3 could be used to identify and characterize important virulence factors that contribute to pathogenesis.

## 1 Data Description

A hypervirulent pathotype of *A. hydrophila,* vAh strains have been responsible for huge losses suffered by the catfish and cyprinid fish industry in USA and China [1,2]. The ability to utilize *myo*-inositol as a growth substrate has been reported to be present in all vAh strains and has not previously been reported among non-vAh strains [3]. This ability has also been linked to contributing to its virulence since *myo-*inositol utilization could allow persistence of *A. hydrophila* strains, and the transcriptional regulator IolR, an important negative regulator of *myo-*inositol pathway has been found to regulate autoaggregation and biofilm formation [3,4]. Therefore, this trait has been used as a key determinant to identify vAh that is also considered to be a primary pathogen [1], unlike typical *A. hydrophila* globally that are considered opportunistic and secondary pathogens [1,2]. *Aeromonas dhakensis* has also been recognized as a significant pathogen in the field of aquaculture. The bacterium is as one of the *Aeromonas* species that is responsible for motile *Aeromonas* septicemia (MAS) [1], and have been reported to be more virulent than some *A. hydrophila* strains [5]. To the author's knowledge, there are no studies that have reported *A. dhakensis* that could utilize *myo*-inositol.

**Fig. 1:**
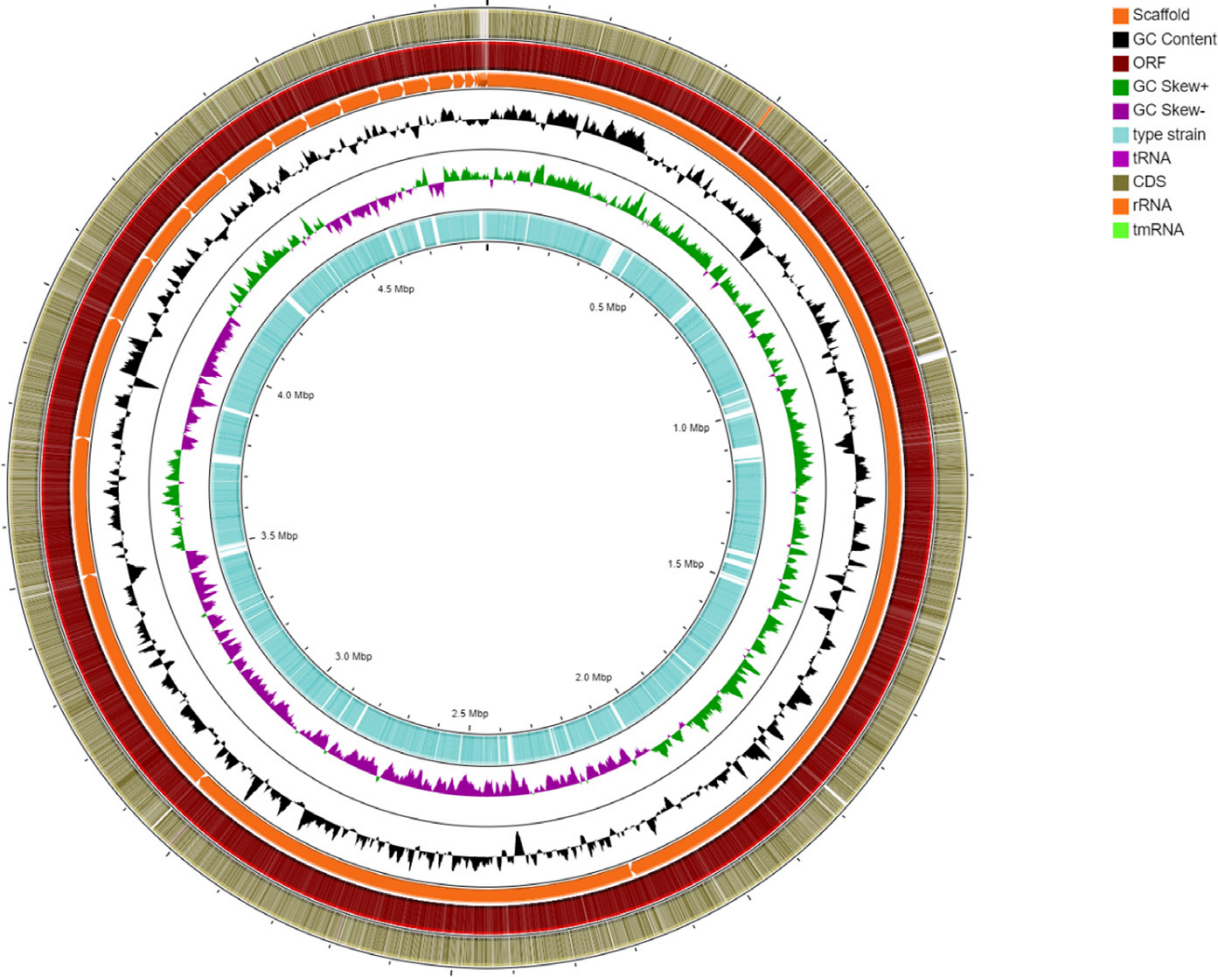
Circular representation of the *A. dhakensis* 1P11S3 genome. From the outside to the center of the diagram, the circles show the following: circle 1, coding region (CDS) (tRNA shown in purple, rRNA shown in orange and tmRNA shown in light green); circle 2, open reading frame (ORF); circle 3, scaffolds; circle 4, GC contents; circle 5, GC skew (G+C shown in green, G-C shown in purple); circle 6, blast comparison with *A. dhakensis* CIP 107500 (T).

The draft genome sequence for *A. hydrophila* 1P11S3 was deposited in GenBank under accession number JADPIC000000000. In total, 4,884,279 bp of total bases were assembled with 43 total scaffolds with a *N*_50_ value of 885,649 with 61.55% G+C content (Table 1, Fig. 1). The genome was predicted to encode 4,391 coding genes, whereas the total non-coding RNA was 182. The major class of carbohydrate-active enzyme was found to be glycosyl transferase (GT; 47.5%), followed by glycoside hydrolases (GH; 25.7%), carbohydrate-binding molecules (CBM; 18.1%), carbohydrate esterases (CE; 7.8%), auxiliary activities (AA; 0.6%) and polysaccharide lyases (PL; 0.2%). The percentage of gene functions present in *A. dhakensis* 1P11S3 in each of six major categories is as follows: general function (11.7%), amino acid transport and metabolism (9.6%), signal transduction mechanisms (7.5%), transcription (7.4%), energy production and conversion (6.2%), and carbohydrate transport and conversion (5.6%). The least categories (0.1%) were extracellular structures, chromatin structure and dynamics, and RNA processing and modification.

**Table 1:** Genome features of *A. dhakensis* 1P11S3.

Attribute	*A. dhakensis* 1P11S3 value
Genome size (bp)	4,884,279
Number of scaffolds	43
N50 (bp)	885649
GC content (%)	61.55
Predicted coding genes	4391
Predicted non-coding RNA	182
GenBank accession	JADPIC000000000
BioSample accession	SAMN16824286
BioProject accession	PRJNA679132

**Fig. 2:**
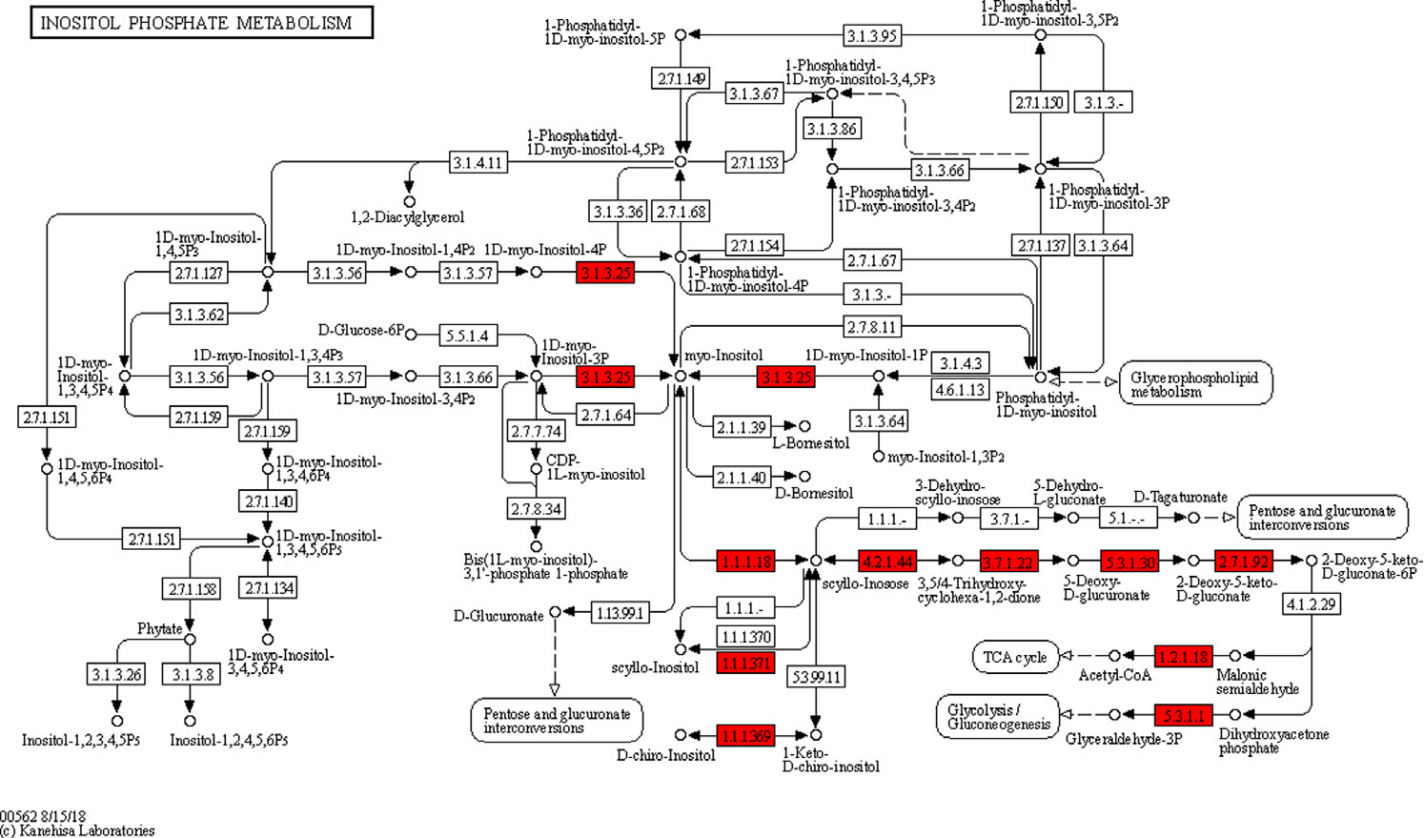
The proposed KEGG pathway for inositol catabolism. Highlighted red boxes indicate the proteins expressed by the *A. dhakensis* 1P11S3 that are involved in the utilization of *myo-*inositol.

KEGG pathway analysis revealed that *A. dhakensis* 1P11S3 has all the proteins necessary for a complete *myo*-inositol metabolism (Fig. 2), in concordance with *A. hydrophila* ML09-119 (vAh) (accession no: CP005966.1, https://www.kegg.jp/kegg-bin/show_pathway?ahy00562), with the extra predicted ability to utilize *scyllo*-inositol, which was apparently not present in *A. hydrophila* ML09-119.

## 2 Experimental Design, Materials and Methods

*Aeromonas dhakensis* 1P11S3 was previously isolated from the spleen of cultured striped catfish (*Pangasianodon hypopthalmus)* in a fish farm in Kelantan, Malaysia, which displayed clinical signs of MAS such as fin and tail rot, hemorrhagic scale and eye, and loss of scale [6]. For the genome analysis, the purified strain of *A. dhakensis* 1P11S3 was inoculated in 1 mL of TSB and incubated overnight at 30°C. Later, DNA extraction was done using a DNA purification kit (Promega, Inc., Madison, WI, USA) according to the manufacturer's protocol. The DNA concentration and quality were determined using Qubit 3.0 Fluorometer (Invitrogen, Carlsbad, CA, USA). Libraries with different indices were multiplexed and loaded onto an Illumina HiSeq instrument according to the manufacturer's instructions (Illumina, San Diego, CA, USA) and the genome was sequenced using a 2 × 150 paired-end sequencing kit. A total of 19,649,882 clean reads (out of 19,688,664 raw reads) were generated and were trimmed of low-quality bases and reads using cutadapt v1.9.1. The final scaffold was then assembled using velvet, and the gaps were filled with SSPACE v3.0 and GapFiller v1-10 [7], [8], [9], [10]. A reference genome, *A. dhakensis* CIP 107500 (type strain) (accession no: CDBH00000000.1), was used to assemble the *A. dhakensis* 1P11S3 genome into scaffolds. Using the default setting of the program tRNAscan-SE v2.0, transfer RNAs (tRNAs) were detected [11]. The coding genes of the bacteria were predicted using the Prodigal v2.6.3 and were annotated with National Center for Biotechnology (NCBI) (https://github.com/hyattpd/Prodigal). Their functions were annotated by Gene Ontology (GO) database, pathways were annotated by Kyoto Encyclopedia of Genes and Genomes (KEGG) and phylogenetic classification of the proteins encoded by genes was done by the Cluster of Orthologous Groups [12], [13], [14].

## CRediT Author Statement

**Mohamad Azzam-Sayuti:** Methodology, Investigation, Formal analysis, Writing – original draft; **Md Yasin Ina-Salwany:** Supervision, Data Curation, Writing – review & editing; **Mohd Zamri-Saad:** Supervision, Data Curation, Writing – review & editing; **Salleh Annas:** Supervision, Writing – review & editing; **Mark R. Liles:** Data Curation, Writing – Review & editing; **Tingbi Xu:** Methodologies, Data Curation; **Mohammad Noor Azmai Amal:** Supervision, Writing – review & editing; **Mohd Termizi Yusof:** Supervision, Data Curation, Writing – review & editing.

## Ethical Statement

Not applicable.

## Declaration of Competing Interest

The authors declared that they have no known competing interests or personal relationships that could have affected the work reported in this paper.

## Appendix Supplementary materials

Image, application 1
